# Validation of a High-Throughput Microfluidic Real-Time PCR for the Detection of Vector-Borne Agents in Wild Birds from the Brazilian Pantanal

**DOI:** 10.3390/pathogens14050491

**Published:** 2025-05-16

**Authors:** Amir Salvador Alabí Córdova, João Batista Pinho, Amanda Garcia Pereira, Clémence Galon, Tiago Valadares Ferreira, Lorena Freitas das Neves, Gabrielly de Oliveira Lopes, Rosangela Zacarias Machado, Sara Moutailler, Marcos Rogério André

**Affiliations:** 1 Vector-Borne Bioagents Laboratory (VBBL), Department of Pathology, Reproduction and One Health, School of Agricultural and Veterinarian Sciences, Sao Paulo State University “Júlio de Mesquita Filho” (FCAV/UNESP), Jaboticabal 14884-900, Brazil; amir.aiabi@unesp.br (A.S.A.C.); garcia.pereira@unesp.br (A.G.P.); lorena.f.neves@unesp.br (L.F.d.N.); gabrielly.lopes@unesp.br (G.d.O.L.); rz.machado@unesp.br (R.Z.M.); 2 Postgraduate Program in Ecology and Biodiversity Conservation, Federal University of Mato Grosso, UFMT, Cuiabá 78060-900, Brazil; joaopinhoufmt@gmail.com (J.B.P.); tiago.biolog@gmail.com (T.V.F.); 3 Agence Nationale de Sécurité Sanitaire de l’Alimentation, de l’Environnement et du Travail (ANSES), Institut National de Recherche en Sciences et Technologies pour l’Environnement et l’Agriculture (INRAE), Ecole Nationale Vétérinaire d’Alfort, UMR BIPAR, Laboratoire de Santé Animale, F-94700 Maisons-Alfort, France; clemence.galon@anses.fr

**Keywords:** Anaplasmataceae, Bartonellaceae, birds, haemosporidians, Onchocercidae, microfluidic real-time PCR

## Abstract

Despite numerous studies on haemosporidians in wild birds from Brazil, the presence of other vector-borne agents (VBA) such as *Anaplasma* spp., *Bartonella* spp., and Onchocercidae filariids in avian hosts remains largely unknown. The low occurrence of these VBAs might be due to the low sensitivity of traditional molecular techniques. The microfluidic real-time PCR assay, known for its high sensitivity, has emerged as a promising method to detect and study the occurrence and diversity of VBAs in both arthropod vectors and vertebrate hosts. To validate previously and standardize newly designed microfluidic real-time PCR protocols, selected positive avian blood DNA samples for *Anaplasma* spp., *Bartonella* spp., haemosporidians, and filariids were used. The molecular occurrence rates for the selected VBAs were 18.2% for *Anaplasma* spp., 0.36% for *Bartonella* spp., 6.2% for *Plasmodium* spp., 4.7% for *Haemoproteus* spp., and 6.5% for Onchocercidae filariids. The *Plasmodium* spp. *cyt*B sequence detected in a *Volatinia jacarina* clustered with *Plasmodium tejerai*, whereas the *Haemoproteus* spp. *cytB* sequence detected in a *Columbina squamata* clustered with *Haemoproteus columbae*. While Onchocercidae filariid *cox-*1 sequences were detected in specimens of *Ramphocelus carbo*, *Turdus amaurocalinus* and *Synallaxis albilora* grouped with *Aproctella* spp., one sequence detected in *R. carbo* was ancestral to the clade comprising *Splendidofilaria* spp. and *Eufilaria* spp. High-throughput microfluidic real-time PCR assay can be used for screening VBAs in avian hosts from South America, but new primers/probe sets should be designed for VBA genotypes present in Brazil.

## 1. Introduction

Although the occurrence and diversity of haemosporidians in wild birds has been extensively investigated in Brazil [1,2,3,4,5,6,7,8], studies on other vector-borne agents (VBA) in avian hosts are limited when it comes to Anaplasmataceae agents, *Bartonella* spp., and filariids.

Anaplasmataceae agents (Order Rickettsiales) stand out among VBA of interest in both animal and human health [9]. Among tick-borne Anaplasmataceae agents, species from three genera are known to infect humans, namely *Anaplasma*, *Ehrlichia*, and ‘*Candidatus* Neoehrlichia’ [10]. Despite this, few studies have investigated the occurrence of Anaplasmataceae in birds [11,12,13,14,15,16]. In Brazil, studies are scarce and reported the occurrence of *Anaplasma* and *Ehrlichia* 16S rRNA genotypes closely related to *Anaplasma phagocytophilum* and *Ehrlichia chaffeensis* [17,18,19,20]. Recently, ‘*Candidatus* Allocryptoplasma spp.’, and *Ehrlichia* spp. closely related to *Ehrlichia* sp. (strain Magellanica) and *Ehrlichia minasensis* were detected in birds from the Brazilian Pantanal [21].

The genus *Bartonella* (order Hyphomicrobiales) comprises fastidious, facultative intracellular alpha-proteobacteria that infect primarily erythrocytes and endothelial cells [22,23]. Its transmission is mainly mediated by arthropod vectors [24,25,26,27,28,29,30,31,32,33], scratches, bodily fluids and bites [23,34]. Currently, there are 42 recognized species (https://www.bacterio.net/genus/bartonella (accessed on 1 May 2024) with 17 species possessing zoonotic potential [35,36]. While *Bartonella henselae*, *Bartonella koehlerae* and *Bartonella tamiae* were detected in wild bird blood samples in the USA [37], *B*. *henselae* and *B*. *machadoae* were detected in wild birds in the Brazilian Pantanal [21,38].

Avian haemosporidians, the most studied blood parasites of birds, are represented by three main protozoa genera, namely *Haemoproteus*, *Leucocytozoon* and *Plasmodium* [39], which are transmitted by blood sucking dipterans [39,40]. Haemosporidians can be found infecting birds worldwide, except in the Antarctica [41]. Although the occurrence and diversity of *Plasmodium* spp. and *Haemoproteus* spp. has been extensively studied in Brazil, the studies were mostly based on conventional PCR assays [6,20], which might have hampered the detection in birds presenting low parasitemia.

Filariids from the Onchocercidae family (subfamilies Dirofilariinae, Onchocercinae, Splendidofilariinae and Lemdaniinae), with a worldwide distribution [40,42], are mainly transmitted by Simuliidae, Culicidae and Ceratopogonidae dipterans. Although microfilariae from *Eulimdana* spp. were detected in blood samples from Charadriiform birds from the United States, Canada and Iceland [43], *Eufilaria delicata* and *Ornithofilaria mavis* microfilariae were detected in Passeriformes from Czech Republic [44], and the majority of the studies reported unidentified filariids in avian blood samples [45,46,47,48].

Microfluidic technology offers a variety of advantages in biology, chemistry and medicine [49]. The microfluidic real-time PCR system has demonstrated to be more efficient on surveillance of neglected disease-causing pathogens. To date, the microfluidic real time PCR has been used to detect a wide variety of tick-borne pathogens [50,51,52], such as *Anaplasma* spp., *Borrelia* spp., *Ehrlichia* spp., *Rickettsia* spp., *Babesia* spp., *Haemoproteus* spp., *Plasmodium* spp., *Leucocytozoon* spp., *Trypanosoma* spp., and *Leptospira* spp. in ticks associated to birds and/or avian populations in France [52,53]. Considering the advantages of microfluidic real-time PCR as its versatility to detect several microorganisms in different samples, such as feces [54], cheese samples [55] and soil [56], simultaneous pathogen detection in multiple independent assays, high sensitivity, low probability of contamination, and time saving when compared to conventional or real-time PCR assays [57]. The present study aimed to detect different vector-borne agents (VBA) in avian blood samples from the Pantanal wetland, central-western Brazil.

## 2. Material and Methods

### 2.1. Sampling

In five days (4 July 2022 to 8 July 2022), 20 mist nets with a 36 mm mesh, 12 m long and 2.5 m of height were placed sideways the trails in the Pantanal region, in the municipalities of Mimoso (−16°12′41″ S, −55°48′31″ W) and São Lourenço (−16°44′33″ S, −55° 33′12″ W). The mist nets were opened instantly after dawn and checked every 30 min for bird removal. The nets were closed at 11:00 A.M. every day. Over 5 days, 300 wild birds were sampled. All the specimens were photographed, identified, and weighed. Each specimen was marked with non-toxic nail-polish and released afterwards. The sampling was approved by the Ethical Commission for the Use of Animal (CEUA 268-21) of the São Paulo State University (FCAV/UNESP). The blood samples were collected from the brachial vein using needles and heparinized capillary tubes. A droplet of blood was used to prepare blood smears for microscopic examination and the remaining blood samples were stored up on FTA cards for molecular analysis. No ticks were found on birds at the sampling time. Blood smears were stained with Rosenfeld staining and analyzed with an Olympus BX43 microscope and photographed with an Olympus camera model DP73 and software cellSens Standard v 1.14.

### 2.2. Molecular Assays

#### 2.2.1. DNA Extraction and PCR Assay for Endogenous Gene

DNA extraction from avian blood samples was performed using the Biopur Mini Spin Plus DNA extraction kit (Mobius Life Science^®^, Paraná, Brazil), following the manufacturer instructions. Blood samples on Whatman^®^ FTA cards were pre-treated as indicated by de Sena Oliveira [58]. DNA concentration, 260/280 and 260/230 ratios were verified in a spectrophotometer (Nanodrop, Thermo Scientific^®^, Waltham, MA, USA). DNA samples were stored at −20 °C until their use in the PCR for endogenous avian *β actin* gene [59], qPCR and the high-throughput microfluidic real-time PCR assays.

#### 2.2.2. Test of Primer and Probe Sets with DNA from Brazilian Strains of Vector-Borne Agents and Avian-Associated Onchocercid Filariids

Among the set of primers and probes used to detect vector-borne agents (VBA) selected for the present study, two primer/probe sets previously designed were selected:

- *Anaplasma* spp. (16S rRNA) [50,51,52];
- *Bartonella* spp. (*ssrA*) [60];

In addition, three primer/probe sets were designed for the following VBA:

- Onchocercid filariids (LSU rRNA);
- *Plasmodium* spp (*cytB*);
- *Haemoproteus* spp. (*cytB*).

Primers and probes were designed by targeting conserved regions from the selected genes and aligning different sequences from the specific gene for each vector-borne agent.

The sets of primers and probes obtained from Defaye, Michelet and Gondard [50,52,60] and those designed for the present study were tested by qPCR assays with positive controls obtained from previous studies from our research group that detected *Bartonella* spp. and Anaplasmataceae agents in Brazilian tropical bird blood samples.

*Dirofilaria immitis* DNA obtained from naturally infected dogs from Brazil (kindly provided by Dr. Norma Labarthe, Oswaldo Cruz Foundation—FIOCRUZ, Rio de Janeiro, RJ, Brazil) and blood DNA sample from a *Ramphocelus carbo* (MIM222) showing microfilariae in the blood smears were used as positive controls in the qPCR assays for onchocercid filariids. *Haemoproteus* sp. DNA obtained from a naturally infected *Columba livia* from Brazil showing *Haemoproteus* spp. trophozoites in blood smears was used as a positive control in the qPCR assays for haemosporidians.

Real-time PCR assays were performed with the kit LightCycler^®^480 Probe Master Mix 1× (Roche Applied Science, Penzberg, Germany), using 200 nM of primers and probes, and 2 µL of DNA in a final volume of 12 µL. The thermal condition was as follows: 95 °C for 5 min, 45 cycles at 95 °C for 10 s and 60 °C for 15 s, and one final cooling cycle at 40 °C for 10 s.

#### 2.2.3. DNA Pre-Amplification for Microfluidic Real-Time PCR

Total DNA was pre-amplified to improve detection of the selected pathogen’s DNA. The Preamp Master Mix (Standard Biotools, South San Francisco, CA, USA) was used according to manufacturer’s instructions. The reaction was performed in a final volume of 5 µL containing 1 µL Preamp Master Mix, 1.25 µL pooled primer mix (200 nM for each primer (Supplementary Table S1), 1.5 µL distilled water and 1.25 µL DNA.

The thermocycling program consisted of one cycle at 95 °C for 2 min, 14 cycles at 95 °C for 15 s and 4 min at 60 °C. At the end of the thermal cycles, the reactions were diluted 1:10 in Milli-Q ultrapure water. All the pre-amplified DNA samples were stored at −20 °C until usage.

#### 2.2.4. High-Throughput Microfluidic Real-Time PCR

To detect DNA from the selected vector-borne agents, the BioMark^TM^ real-time PCR system (Standard Biotools, South San Francisco, CA, USA) was used for High-throughput microfluidic real-time PCR, using 48.48 dynamic arrays.

The chips distributed 48 PCR assays (primers and probe) and 48 samples into single chambers. The on-chip microfluidic assembled PCR reactions in specific chambers previously to thermal cycling. Primers and probes used in the microfluidic real-time PCR assays are listed in Supplementary Table S1.

Amplifications were performed using 6-carboxyfluorescein (FAM) and black hole quencher (BHQ1) labeled Taqman^®^ probes with Taqman^®^ Gene expression master mix according to the manufacturer’s instructions (Applied biosystems, Waltham, MA, USA).

The thermal cycling conditions comprised 2 min of 50 °C, 10 min at 95 °C, followed by 40 cycles of 2-step amplification of 15 s at 95 °C and 1 min at 60 °C. Data were collected on the BioMark^TM^ Real-Time PCR System and analyzed using the Fluidigm Real-Time PCR Analysis v 4.7.1 software to obtain crossing point (CP) values. Samples were run along with one negative water control per chip, and one control for PCR inhibition using *Escherichia coli* DNA.

#### 2.2.5. Validation by PCR and Sanger Sequencing

Conventional PCR assays using primers targeting molecular markers distinct from those of the BioMark^TM^ system were used to confirm the presence of the selected VBA DNA in bird blood samples (Table 1). For validation of the microfluidic real-time PCR assays, only samples showing low Cq values (<30) were subjected to conventional PCR assays. Amplicons were sequenced by Eurofins MWG Operon (Ebersberg, Germany) and then edited with BioEdit v7.2.5 software [61]. BLASTn [62] the obtained sequences with those previously deposited in GenBank database.

#### 2.2.6. Phylogenetic Analyses

The obtained sequences from conventional PCR assays were aligned via MAFFT [79] with homologous sequences retrieved from the GenBank database. The alignment saved as “FASTA” was used to infer the evolutionary model. Phylogenetic inferences were performed using the IQtree online version (http://iqtree.cibiv.univie.ac.at/ (accessed on 1 May 2025)) [80]. Clade supports for maximum likelihood analyses (ML) were evaluated using bootstraps analyses [81] of 100 repetitions. The phylogenetic tree was edited using Figtree V1.4.4 (http://tree.bio.ed.ac.uk/software/figtree/ (accessed on 1 May 2025) [82].

## 3. Results

### 3.1. DNA Extraction and PCR Assay for Avian β Actin Endogenous Gene

The extracted DNA samples presented an average concentration of 7.78 ng/uL and ratios 260/280 of 3.10 and 260/230 of −0.96. In the PCR assay, 275/282 (97.5%) avian DNA blood samples showed a positive result for the avian *β-actin* gene. Negative samples in the *β-actin* gene-based PCR assay were excluded for the subsequent molecular analyses.

### 3.2. Testing of Primer and Probe Sets Designed for Microfluidic Real-Time PCR by qPCR Assay

The designed primers/probe sets previously designed for *Anaplasma* spp. (16S rRNA) and *Bartonella* spp. (ssrA) [60] were able to detect genotypes of *Anaplasma* spp., ‘*Candidatus* Allocryptoplasma spp.’, and *Bartonella* spp. previously detected in avian blood DNA samples from Brazil [21,38]. Then, they were conserved to screen Brazilian samples through the high-throughput microfluidic real-time PCR system.

When testing the positive samples previously obtained from bird blood samples from Brazil, the following results were shown:

- *Anaplasma* spp./‘*Candidatus* Allocryptoplasma spp.’ were detected in five (100%) out of five samples in the qPCR assay targeting the 16S rRNA for *Anaplasma* spp./‘*Candidatus* Allocryptoplasma spp.’
- *Bartonella* spp., was detected in three (60%) out of five samples in the qPCR assay targeting the *ssr*A gene.

### 3.3. Primer and Probe Sets Tested in High-Throughput Microfluidic Real-Time PCR Assay

The following primers and probes were used in the high-throughput microfluidic real-time PCR assay:

(i.) designed in previous studies for *Anaplasma* spp. (16S rRNA), *Bartonella* spp. (ssrA), Apicomplexa (18S rRNA), *Aegyptianella pullorum* (groEL), *Rickettsia* spp. (gltA), *Rickettsia africae* (sca1), *B. vinsonii berkhoffii* (16S-23S rRNA ITS) and *Hepatozoon* spp. (18S rRNA) [60] *Borrelia* spp. (23S rRNA), *Rickettsia rickettsii* (23S-5S rRNA ITS), *Borrelia burgdorferi* s.s. (rpoB), *Borrelia garinii* (rpoB), *Borrelia valaisiana* (ospA), *B. henselae* (pap-31), *A. phagocytophilum* (msp2), *E. chaffeensis* (dsb), *Rickettsia massiliae* (23S-5S rRNA ITS), Spotted Fever Group *Rickettsia* spp. (gltA), *Babesia vogeli* (hsp70) [49], and *Trypanosoma* spp. (18S rRNA) [52].
(ii.) designed in the present study for *Ehrlichia* spp. (groEL), *Borrelia* spp. (flaB), *Trypanosoma* spp. (18S rRNA), *Plasmodium* spp. (cytB), *Haemoproteus* spp. (cytB), *Leucocytozoon* spp (ssRNA), *Aproctella* spp. (LSU rRNA), *Chandlerella* spp. (cox1), *Eufilaria* spp. (LSU rRNA).

When testing the 36 bird blood DNA samples from Brazil positive to VBA obtained in previous studies [21,38] the following occurrences for several BVA were obtained:

- Eleven (30.5%) out of these thirty-six bird blood DNA samples were positive to different VBA in high-throughput microfluidic real-time PCR assay (Supplementary Material Table S2).
- Five (38.5%) out of thirteen bird blood DNA samples previously positive to *Anaplasma* spp. and ‘*Candidatus* Allocryptoplasma spp.’ (obtained by our research group in a previous study [21] were also positive in the microfluidic real-time PCR using primers previously described by Gondard [60].
- Four (44.4%) out of nine positive bird blood DNA samples previously positive to *Bartonella* spp. (obtained by our research group in a previous study [38].

The primers/probe sets designed in previous studies [60] were able to amplify *Anaplasma* spp., ‘*Candidatus* Allocryptoplasma spp.’, *Bartonella* spp. and *Bartonella machadoae*. All samples were negative in microfluidic real-time PCR assays for the remaining vector-borne agents tested.

### 3.4. High-Throughput Microfluidic Real-Time PCR Assay

The molecular occurrence for the selected VBA was as follows:

- *Anaplasma* spp. 18.2% (50/275);
- *Bartonella* spp. 0.36% (1/275);
- *Plasmodium* spp. 6.2% (17/275);
- *Haemoproteus* spp. 5.09% (14/275);
- Onchocercid filariids 6.5% (18/275)

The following single infections were observed (Supplementary Material Table S2) in the microfluidic real-time PCR assays:

- *Anaplasma* spp. 12.72% (35/275);
- *Bartonella* spp. 0.36% (1/275);
- *Plasmodium* spp. 3.63% (10/275);
- *Haemoproteus* spp. 2.90% (8/275);
- Onchocercidae filariids 2.90% (8/275).

The following co-infections were observed in the microfluidic real-time PCR assays (Supplementary Material Table S3):

- *Anaplasma* spp. + filariids 3.30% (9/275);
- *Anaplasma* spp. + *Plasmodium* spp. 1.09% (3/275);
- *Anaplasma* spp. + *Haemoproteus* spp. 1.09% (3/275);
- *Plasmodium* spp. + *Haemoproteus* spp. 1.09% (3/275);
- *Plasmodium* spp. + Onchocercidae filariids 0.36% (1/275).

### 3.5. Validation of Microfluidic Real-Time PCR Results by Conventional PCR Assays and Sanger Sequencing

Out of 50 positive samples for *Anaplasma* spp. in the microfluidic real-time PCR, 13 (26%) samples were subjected to conventional PCR assays targeting the 16S rRNA gene and 23S-5S intergenic region (ITS) (Saolou4, Saolou15, Saolou61, Saolou82, Saolou83, Saolou93, Saolou94, Saolou103, Saolou115, Saolou125, Saolou126, MIM176, MIM243). One sample *(Eucometis pennicillata*) was positive (Saolou 82 Genbank accession number PQ450485) showing 100% identity and a query cover of 90% with *Anaplasma* spp. previously detected in *Basileuterus flaveolus* from Brazil (PP417936).

Out of 30 samples positive for hemosporidians (*Haemoproteus* spp. and *Plasmodium* spp.), 12 (38.7%) were subjected to a conventional PCR targeting the *cytB* gene for *Haemoproteus* spp., *Plasmodium* spp., and *Leucocytozoon* spp. (Saolou48, MIM154, MIM218, MIM230, MIM236, MIM242, MIM243, MIM250, MIM289, MIM292, MIM296, MIM298). As a result, five samples were positive, and two readable sequences were obtained: sample MIM250 (*Columbina squammata*) (PQ450488) showed a 100% identity (100% query cover) with *Haemoproteus* spp. (KP686107) and sample MIM292 (*Volatinia jacarina*) (PQ450487) showed 100% identity (97% query cover) with *Plasmodium* spp. (KY304999).

Out of 23 samples positive for Onchocercid filariids, 15 (65.2%) were subjected to conventional PCR assays targeting the *cox-1*, 12S RNA and 28S rRNA genes (Saolou30, Saolou32, Saolou41, Saolou55, Saolou67, Saolou81, Saolou85, Saolou94, Saolou108, Saolou114, Saolou126, 179, 420, 441 and 497). As a result, 10 samples were positive in the *cox-1*-based conventional PCR, and nine sequences were obtained.

Samples Saolou30 (*Ramphocelus carbo*) (PQ452771), Saolou32 (*Ramphocelus carbo*) (PQ452776), Saolou41 (*Ramphocelus carbo*) (PQ452777), Saolou55 (*Ramphocelus carbo*) (PQ452778), Saolou67 (*Turdus amaurochalinus*) (PQ452774), Saolou114 (*Synallaxis albilora*) (PQ452775) and Saolou126 (*Ramphocelus carbo*) (PQ452772) showed 97.8–100% identity (100% query cover) with *Aproctella* spp. (FR823331). The sample Saolou94 (*Ramphocelus carbo*) (PQ452773) showed 92.2% identity (99% of query cover) with *Eufilaria sylviae* (MT800771).

Although one blood sample from *Fomicivora rufa* was positive in the microfluidic PCR for *Bartonella* spp., no readable sequences were obtained in the conventional PCR assays based on the *Bartonella* 16-23S rRNA intergenic region (ITS), and *gltA*, *groEL*, *fts*Z, *nuoG*, *ribC*, *pap31*, and *rpoB* genes.

### 3.6. Phylogenetic Positioning of the Obtained Sequences

**Figure 1:**
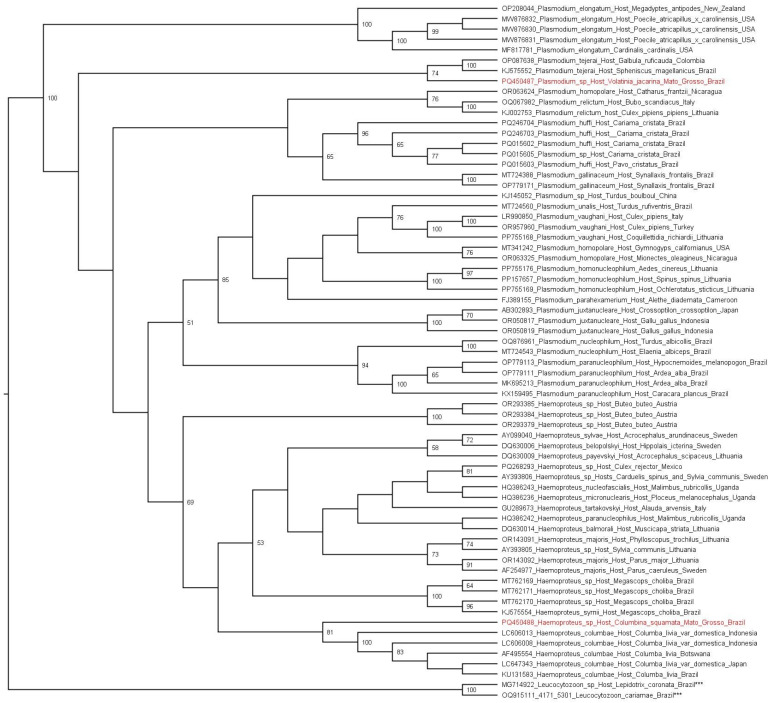
Phylogenetic analysis inferred by maximum likelihood method (GTR + F + I + G4 evolutionary model) based on a 328 bp alignment of the *cytB* gene containing 67 haemosporidian homologous sequences (*Plasmodium* spp. and *Haemoproteus* spp.). *Leucocytozoon* species (MG714922, MG209789 and OQ915111) were used as outgroups. Sequences obtained in the present study are highlighted in red and the outgroups are indicated with “***”. Only the bootstraps with values of 50 or more are shown.

The phylogenetic analysis based on the *cytB* gene (alignment of 328 bp) of haemosporidians inferred by maximum likelihood method and GTR + F + I + G4 evolutionary model showed two major clades: (i) the avian related *Plasmodium* species clade, in which sequence of *Plasmodium* spp. detected in the present study (Genbank accession number PQ450487) was positioned within a clade containing sequences of *Plasmodium tejerai* from Colombia (OP087638) and Brazil (KJ57552); and (ii) the *Haemoproteus* species clade, in which the sequence from the present study (Genbank accession number PQ450488) was positioned with *Haemoproteus columbae* sequences from Indonesia (LC606013, LC606008), Botswana (AF495554), Japan (LC647343) and Brazil (KU131583) (Figure 1).

**Figure 2:**
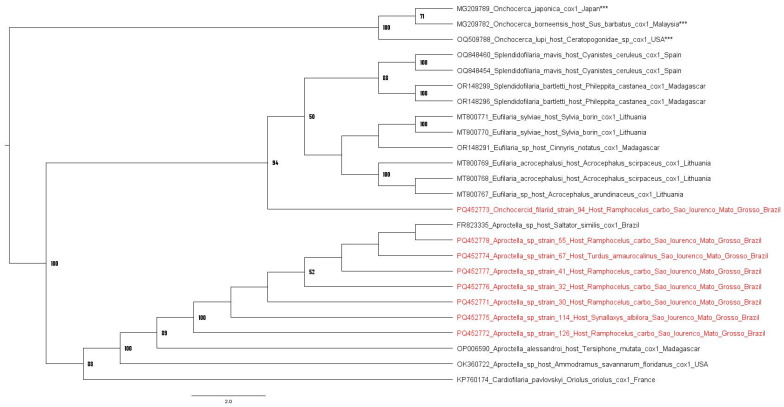
Phylogenetic analysis inferred by maximum likelihood method (GTR + F + I + G4) evolutionary model based on a 660 bp alignment of the *cox*-*1* gene of 25 onchocercid filariid homologous sequences (*Aproctella* spp., *Eufilaria* spp., *Cardiofilaria* spp., *Splendidofilaria* spp.). *Onchocerca* species (MG209782, MG209789 and OQ509788) were used as outgroups. Sequences obtained in present study are highlighted in red and the outgroups with “***”. Only the bootstraps with values of 50 or more were shown.

The phylogenetic analyses based on the *cox*-*1* gene of onchocercid filariids (alignment of 673 bp) inferred by maximum likelihood method and GTR + F + I + G4 evolutionary model showed six different clades: (i) the first clade grouped *Splendidofilaria* species sequences; (ii) the second clade contained *Eufilaria* species sequences; (iii) the third clade contained a sequence of the present study obtained from *Ramphocelus carbo* (PQ452773) from Sao Lourenço, Mato Grosso, as an ancestral branch to *Eufilaria* and *Splendidofilaria*; (iv) the fourth clade composed by *Aproctella* species from Madagascar (OP006590), USA (OK360722) and Brazil (FR823335). The majority of the sequences obtained in the present study were positioned within the *Aproctella* clade, namely PQ452778 (*Ramphocelus carbo*), PQ452774 (*Turdus amaurocalinus*), PQ452777 (*Ramphocelus carbo*), PQ452776 (*Ramphocelus carbo*), PQ452771 (*Ramphocelus carbo*), PQ4552772 (*Ramphocelus carbo*) and PQ452775 (*Synallaxis albilora*), which were closely related to a sequence previously detected in *Saltator similis* from Brazil (FR823335), and (v) the fifth clade is composed by a *Cardiofilaria paylovski* from France (Figure 2).

When examining the blood smears of the positive samples for onchocercid filariids and haemosporidians, onchocercid microfilariae were found in blood smears from two specimens of *R. carbo* (SaoLou 32 and SaoLou 41) that showed to be positive for *Aproctella* spp. Microfilariae were also found in the blood smear of one specimen (SaoLou85) of *Saltator coerulescens* that was positive in the microfluidic real-time PCR but negative in the conventional PCR targeting the *cox-1* gene (Supplementary Material Figure S1).

## 4. Discussion

To the best of the authors’ knowledge, this is the first time that high-throughput microfluidic real-time PCR assay was applied to detect vector-borne pathogen DNA in avian blood samples collected in the Americas. In the present study, multiple primers and probes sets were designed to be implemented for the detection of vector-borne agents (*Plasmodium* spp., *Haemoproteus* spp., Onchocercid filariids) in DNA blood samples from birds sampled in the Pantanal wetland, Mato Grosso state, central–western Brazil. In previous studies, high-throughput microfluidic real-time was used to detect DNA from selected pathogens in ticks associated to birds and avian blood samples from France [50,51,52]. The use of this technique ensures multiple independent assays [57] and efficiency in the surveillance of neglected pathogens [50,51,52].

Primers and probes designed in previous studies [60] and targeting the 16S rRNA gene of *Anaplasma* agents and *ssr*A genes of *Bartonella* spp. agents were able to detect DNA of Anaplasmataceae/Bartonellaceae agents in bird blood DNA samples from Brazil that showed to be previously positive for *Anaplasma* spp., ‘*Candidatus* Allocryptoplasma spp.’ and *Bartonella* spp. [21,38]. Indeed, although the previously described primers were designed to catch the common genotypes circulating in Europe, which are genetically distinct from those found in birds and wildlife from Brazil, they were able to catch some novel *Bartonella* and *Anaplasma* and ‘*Candidatus* Allocryptoplasma spp.’ recently reported in Brazil.

While microfluidic real-time PCR revealed a positivity rate of 18.2% for *Anaplasma*, *Candidatus* Allocryptoplasma spp. among birds sampled in the Pantanal of Mato Grosso state, our previous study performed in the states of Mato Grosso and Mato Grosso do Sul reported an occurrence of 7.9% using both real-time quantitative (q)PCR and conventional PCR assays [21,38]. One *Anaplasma* spp. 23S-5S rRNA (ITS) sequence showed 100% identity and a query cover of 90% with *Anaplasma* spp. previously detected in *B. flaveolus* from Brazil.

This finding validates the specificity of the primers/probe used for *Anaplasma* spp. previously designed by Gondard [60], showing that they can catch *Anaplasma* species that so far have only been detected in Brazil [21,38].

The topology of phylogenetic analysis based on the haemosporidians *cytB* gene performed herein corroborated previously reported results [6,83,84,85,86], displaying the separation between *Plasmodium* and *Haemoproteus* species. While the *Plasmodium* spp. *cyt*B sequence detected in a specimen of *V. jacarina* clustered with *Plasmodium tejerai*, the *Haemoproteus* spp. *cy*tB sequence detected in a specimen of *C. squamata* clustered with *Haemoproteus columbae* obtained from other Columbiformes. These findings demonstrated that the primers/probes designed herein are able to catch *Haemoproteus*/*Plasmodium* lineages from Brazil [83,84]

The topology of phylogenetic analysis based on the *cox*-1 gene of onchocercid filariids presented herein showed similar results to those presented by Binkienė and Hayashi [79,86]. The Onchocercid filariid *cox*-*1* sequence detected in a specimen of *Ramphocelus carbo* showed to be ancestral to the clade comprising *Splendidofilaria* spp. and *Eufilaria* spp. On the other hand, Onchocercid filariid *cox*-*1* sequences detected in specimens of *R. carbo*, *T. amaurocalinus* and *S. albilora* grouped with *Aproctella* spp. Therefore, the primers and probe designed in the present study were able to detect at least two distinct avian-associated Onchocercidae filariid species.

Unfortunately, no readable sequence was obtained from positive samples for *Bartonella* spp., precluding assessing the *Bartonella* species detected in birds sampled in this study. This finding could be related to the expected low *Bartonella* bacteremia in birds. Instead, a single sequence of *Bartonella machadoae* was obtained from a *F. rufa* blood sample used as a positive control [21] (accession number PV083549).

Despite their potential presence, the low occurrence rates of certain vector-borne agents (such as *Bartonella* spp.) in avian hosts are usually impacted by the limitations of traditional molecular detection methods. The use of molecular techniques showing higher sensitivity and specificity is paramount in unravelling the disease dynamics in non-usual hosts. Although *Bartonella* spp. has classically been associated with mammals [35,87,88,89,90,91,92,93], the role of birds as potential hosts for these agents has been neglected due to limitations of traditional molecular approaches used until now.

Although the microfluidic real-time PCR assay has shown promise in detecting a wide range of vector-borne agents in South American avian hosts, there are potential challenges that must be taken into account when developing new primers and probes for the specific genotypes present in Brazil, including the following: (i) dealing with very low parasitemia of the studied agents in bird blood samples, although the pre-amplification step helps to increase the detection yield of the DNA template [94]; (ii) being aware that distinct genotypes of VBA occur in South America, which may hamper the use of primers and probes previously designed to “catch” strains and variants that occur in other regions of the world; and (iii) that the occurrence of cross-amplification of avian host DNA using primers/probes works perfectly on mammal biological DNA samples. Therefore, caution is needed when using previously designed primer/probe sets, since the accuracy and generalizability of the assay across different regions and hosts may not be as optimal as expected.

Finally, taking into account the environmental factors and genetic diversity of VBA in birds in South America, it is paramount to validate previously designed primers/probes with positive controls representing the genotypes that have been found in a certain geographical region.

## 5. Conclusions

The high-throughput microfluidic real-time PCR assay based on previously designed primers for Europe and the Caribbean regions was able to detect several positive samples to vector-borne agents from the present study samples, suggesting the field samples from different regions should be tested first with the primers designed for the European and Caribbean regions and, in case of variability due to different genotypes, new primers and probes should be designed. High-throughput microfluidic real-time PCR assay can be used to screen *Anaplasma* spp., *Bartonella* spp., *Plasmodium* spp., *Haemoproteus* spp., and Onchocercidae filariids associated with avian hosts from South America. In addition, this molecular approach can assist in understanding the epidemiological and ecological dynamics of vector-borne agents among birds in the Pantanal, the largest wetland in South America.

## Figures and Tables

**Table 1:** Primer sequences, amplicon sizes, and target genes used in conventional PCR assays for validation of the high-throughput microfluidic real-time PCR for selected vector-borne agents in avian blood samples from the Brazilian Pantanal.

Agents	Sequences (5′-3′)	Size (bp)	Molecular Marker	Reference
*Anaplasma* spp.*/Ehrlichia* spp.	EHR1 GAACGAACGCTGGCGGCAAGC EHR2 AGTA(T/C) CG(A/G) ACCAGATAGCCGC EHR3 TGCATAGGAATCTACCTAGTAG EHR2 AGTA(T/C) CG(A/G) ACCAGATAGCCGC	693 592	16S rRNA	[63]
*Anaplasma* spp.	GE2F2 GTTAGTGGCAGACGGGTGAGT AE4-Fw GTACCYAYAGAAGAAGTCCCGGCA AE-Rv RCACCAGCTTCGAGTTAAGCCAAT	800	16S rRNA	[64,65,66]
*Anaplasma* spp.	ITSiF ATACCTCTGGTGTACCAGTTG ITSiR TTAACTTCCGGGTTCGGAATG	300	ITS 23S-5S	[67]
*Bartonella* spp.	P-bhenfa TCTTCGTTTCTCTTTCTTCA P-benr1 CAAGCGCGCGCTCTAACC N-bhenf1a GATGATCCCAAGCCTTCTGGC N-bhenr AACCAACTGAGCTACAAGCC	Depends on the species	16S-23S rRNA (ITS)	[68]
	BhCS.781p F GGGGACCAGCTCATGGTGG BhCS.1137n R AATGCAAAAAGAACAGTAAACA	380–400	*glt* *A*	[69]
	CS443f GCTATGTCTGCATTCTATCA CS1210r GATCYTCAATCATTTCTTTCCA	767	*glt* *A*	[24]
	ftsZ F CATATGGTTTTCATTACTGCYGGTATGG ftsZ R TTCTTCGCGAATACGATTAGCAGCTTC	515	*fts* *Z*	[70]
	groEL F GGAAAAAGTGGGCAATGAAG groEL R TCCTTTAACGGTCAACGCATT	752	*gro* *EL*	[70]
	nuoG F GGCGTGATTGTTCTCGTTA nuoG R CACGACCACGGCTATCAAT	346	nuoG	[71]
	165s GACTTCTGTTATCGCTTTGATTT 688as CACCACCAGCAAMATAAGGCAT	564	*pap*31	[72]
	1400F CGCATTGGCTTACTTCGTATG 2300R GTAGACTGATTAGAACGCTG	825	*rpoB*	[73]
	Barton-1 TAACCGATATTGGTTGTGTTGAAG Barton-2 TAAAGCTAGAAAGTCTGGCAACATAACG	585–588	*ribC*	[74]
	321s AGATGATGATCCCAAGCCTTCTGGCG 938as TGTTCTYACAACAATGATGATG	453–717	16S-23S rRNA (ITS)	[75]
Haemosporidians (*Haemoproteus* spp., *Plasmodium* spp. and *Leucocytozoon* spp.)	HaemNFI-cytB-F CATATATTAAGAGAAITATGGAG HaemNR3-cytB-R ATAGAAAGATAAGAAATACCATTC HaemF-cytB ATGGTGCTTTCGATATATGCATG HaemR2-CYTb GCATTATCTGGATGTGATAATGGT HaemFL-cytB ATGGTGTTTTAGATACTTACATT HaemR2L-cytB CATTATCTGGATGAGATAATGGIGC	660 480 478	*cytB*	[76]
Onchocercid filariids	NTF-*cox*F TGATTGGTGGTTTTGGTAA NTR-*cox*R ATAAGTACGAGTATCAATATC	650	*cox-1*	[77]
Onchocercidae	988F CTCAAAGATTAAGCCATGC 1912R TTTACGGTCAGAACTAGGG	998	12S rRNA	[78]
Onchocercidae	Nematode 1 GCGGAGGAAAAGAAACTAA Nematode 2 ATCCGTGTTTCAAGACGGG	855	28S rRNA	[78]

## Data Availability

The sequences generated and analyzed during the present study were submitted in the NCBI Genbank (https://www.ncbi.nlm.nih.gov/genbank/. Sequences can be accessed by the following accession numbers: PQ450485, PQ450488, PQ450487, PQ452771, PQ452776, PQ452777, PQ452778, PQ452774, PQ452775, PQ452772, FR823331, PQ452773 and PV083549.

## Supplementary Materials

The following supporting information can be downloaded at https://www.mdpi.com/article/10.3390/pathogens14050491/s1; Table S1: Description of primers (F = forward; R = reverse), probes (P) and target genes used in the microfluidic real-time PCR assays for the selected vector-borne agents in avian blood samples from the Brazilian Pantanal; Table S2: Results of High-Throughput Microfluidic Real-Time PCR assay with positive controls obtained from neotropical birds sampled in Pantanal wetland, Mato Grosso state, central-western Brazil [21,38]; Table S3: Results of High-Throughput Microfluidic real-time PCR showing co-positivity for selected vector-borne agents in neotropical birds sampled in Pantanal wetland, Mato Grosso state, central-western Brazil. Figure S1: Microfilariae detected in avian sample blood smears. (A) SaoLou30 and (B) SaoLou41 were identified as *Aproctella* spp. due to sequencing and phylogenetic inference of the *cox-1* gene.
