# Supplementary material for: Validation of a High-Throughput Microfluidic Real-Time PCR for the Detection of Vector-Borne Agents in Wild Birds from the Brazilian Pantanal
Source: Pathogens. 2025 May 16;14(5):491. doi: 10.3390/pathogens14050491 (PMC12114467; doi:10.3390/pathogens14050491)
Supplement: Supplementary file 1 [file pathogens-14-00491-s001.zip › pathogens-3592589-supplementary.pdf]

Supplementary Materials

Supplementary Material - Table S1. Description of primers (F= forward; R= reverse), probes (P) and target genes used in the microfluidic real-time PCR assays for the selected vector-borne agents in avian blood samples from the Brazilian Pantanal.

Agents	Sequences (5'-3')	Length (bp)	Target Gene	Reference
Anaplasma spp.	F: CTTAGGGTTGTAAAACTCTTTCAG<br>R: CTTTAACTTACCAAACCGCCTAC<br>P: ATGCCCTTTACGCCCAATAATTCCGAACA	160	16S rRNA	[59]
Anaplasma phagocytophilum	F: GCTATGGAAGGCAGTGTTGG<br>R: GTCTTGAAGCGCTCGTAACC<br>P: AATCTCAAGCTCAACCCTGGCACCAC	77	*msp*2	[49]
Ehrlichia spp.	F: GCAACGCGAAAAACCTTACCA<br>R: AGCCATGCAGCACCTGTGT<br>P: AAGGTCCAGCCAAACTGACTCTTCCG	98	16S rRNA	[59]
Ehrlichia chaffeensis	F: TATTGCTAATTACCCTCAAAAAGTC<br>R: GAGCTATCCTCAAGTTCAGATTT<br>P: ATTGACCTCCTAACTAGAGGGCAAGCA	117	*dsb*	[59]
Aegyptianella pullorum	F: AGCCAGTATTATCGCTCAAGG<br>R: GCCTCACGTGCCTTCATAAC	165	*groEL*	[59]

	P: TGCTTCTCAGTGTAACGACAGGGTTGG			
Ehrlichia spp. [AA]	F: GTGGTGGATTACATGTAGCAG<br>R: CTTAGCAGTACCCAATTCAGC<br>P: CAGTGAAGGCTCCAGGATTTGGTGATAGAA	164	*groEL*	Present study
Bartonella spp.	F: CGTTATCGGGCTAAATGAGTAG<br>R: ACCCCGCTTAAACCTGCGA<br>P: TTGCAAATGACAACTATGCGGAAGCACGTC	118	*ssrA*	[59]
Bartonella hensellae	F: CCGCTGATCGCATTATGCCT<br>R: AGCGATTTCTGCATCATCTGCT<br>P: ATGTTGCTGGTGGTGTTTCCTATGCAC	107	*pap*31	[49]
Bartonella vinsonii berkhoffi	F: GGAATTGCTTAACCCACTGTTG<br>R: CCTTATTGATTTAGATCTGATGGG<br>P: AGAAACTCCCGCCTTTATGAGAGAAATCTCT	141	16-23S rRNA (ITS)	[59]
Hepatozoon spp.	F: ATTGGCTTACCGTGGCAGTG<br>R: AAAGCATTTTAACTGCCTTGTATTG<br>P: ACGGTTAACGGGGGATTAGGGTTCGAT	175	18S rRNA	[49]
Apicomplexa protozoa	F: TGAACGAGGAATGCCTAGTATG<br>R: CACCGGATCACTCGATCGG<br>P: TAGGAGCGACGGGCGGTGTGTAC	104	18S rRNA	[59]

Trypanosoma spp.	F: GTAATTCCAGCTCCAAAAGCG<br>R: TCAGGAAGGAACCACTCCC<br>P: ACCTCAAGGGCATGGGTCACCAATCC	178	18S rRNA	[51]
Babesia vogeli	F: TCACTGTGCCTGCGTACTTC<br>R: TGATACGCATGACGTTGAGAC<br>P: AACGACTCCCAGCGCCAGGCCAC	87	*hsp*70	[51]
Borrelia garinii	F: TGGCCGAACTTACCCACAAAA<br>R: ACATCTCTTACTTCAAATCCTGC<br>P: TCTATCTCTTGAAAGTCCCCCTGGTCC	88	*rpo*B	[49]
Borrelia valaisiana	F: ACTCACAAATGACAGATGCTGAA<br>R: GCTTGCTTAAAGTAACAGTACCT<br>P: TCCGCCTACAAGATTTCCTGGAAGCTT	135	*osp*A	[49]
Borrelia sp.	F: GAGTCTTAAAAGGGCGATTTAGT<br>R: CTTCAGCCTGGCCATAAATAG<br>P: AGATGTGGTAGACCCGAAGCCGAGT	73	23S rRNA	[49]
Borrelia burgdorferi sensu stricto	F: GCTTACTCACAAAAGGCGTCTT<br>R: GCACATCTCTTACTTCAAATCCT<br>P: AATGCTCTTGGACCAGGAGGACTTTCA	83	*rpo*B	[49]
Rickettsia spp.	F: GTCGCAAATGTTCACGGTACTT<br>R: TCTTCGTGCATTTCTTTCCATTG	78	*gltA*	[59]

	P: TGCAATAGCAAGAACCGTAGGCTGGATG			
Rickettsia rickettsii	F: TCTACTCACAAAGTTATCAGGTTAA<br>R: CCTACGATACTCAGCAAAATAATTT<br>P: TCGCTGGATATCGTTGCAGGACTACAG	124	23S-5S rRNA ITS	[49]
Rickettsia massiliae	F: GTTATTGCATCACTAATGTTATACTG<br>R: GTTAATGTTGTTGCACGACTCAA<br>P: AGCCCCGCCACGATATCTAGCAAAAA	128	23S-5S rRNA ITS	[59]
Rickettsia africae	F: GATACGACAAGTACCTCGCAG<br>R: GGATTATATACTTTAGGTTCGTTAG<br>P: CAGATAGGAACAGTAATTGTAACGGAACCAG	122	Sca1	[59]
Spotted fever group *Rickettsia*	F: CCTTTTGTAGCTCTTCTCATCC<br>R: GCGATGGTAGGTATCTTAGCAA<br>P: TGGCTATTATGCTTGCGGCTGTCGGT	145	*gltA*	[49]
Borrelia spp. [AA]	F: GCTGAAGAGCTTGGAATGCAG<br>R: GCAATTGCTTCATCCTGATTTG<br>P: CCTGCAAAAATTAACACACCAGCATCTCTATC	116	*fla*B	Present study
Haemoproteus spp. [AA]	F: CTTGGGGTCAAATGAGTTTCTG<br>R: GGTAGGGTCACTAACAATATATC<br>P: CACCACAAATCCATGAGACTAATCCAGGTATA	240	*cyt*B	Present study

Plasmodium spp. [AA]	F: TACCTGGTCTTGTCTCATGG<br>R: CCCTAAAGGATTTGTGCTACC<br>P: TTTGTGGTGGATATCTTGTAAGCGACCCAAC	339	*cytB*	Present study
Trypanosoma spp. [AA]	F: TGAACCAAAGGGACGCTCTC<br>R: CTTCTGGGTGTTACTGCCG<br>P: CTGTTCCGGCGATGGGGCAACTC	112	18S rRNA	Present study
Leucocytozoon spp. [AA]	F: GTAATGTAGAAACTGCGAACGG<br>R: CTTATACGTGTCGCTTCTTTGT<br>P: TTAGGACTCCCCACTTGTCTTTTTCTTGAAA	177	*ssRNA*	Present study
Aproctella spp [AA]	F: GCATATTTTTACACCCAAAGTCC<br>R: CTAAAGTGTAACCGTACCGTAAC<br>P: CTTGAGCGGGGCTACAGTCCATAGAAG	98	LSU rRNA	Present study
Eufilaria spp. [AA]	F: ACCCAAAGTCCCCCTTGAG<br>R: CGTAACGTTTTTCACCCGTAC<br>P: CGAGGCCATTATCCATAGAAGGTGCTAG	72	LSU rRNA	Present study
Chandlerella spp. [AA]	F: TATTGGGATCTCCTGAAATGGC<br>R: AGGCTGACCTTCAACACTTAATG_<br>P: TGGATAAAAAGTTCAACTTCTACCAGGGCCA	158	*cox-1*	Present study

[AA]: Stands for primers and probes designed in the present study.

Supplementary Material - Table S2. Results of High-Throughput Microfluidic Real-Time PCR assay with positive controls obtained from neotropical birds sampled in Pantanal wetland, Mato Grosso state, central-western Brazil (Alabí-Córdova et al., 2024 a;b).

Positive samples on microfluidic PCR	Scientific name	*Anaplasma* spp* 16S rRNA	*Bartonella* spp. * *ssr*A	*Plasmodium* spp.[AA] *cyt*B	*Haemoproteus* spp.[AA] *cyt*B	Onchocercidae filariids [AA] LSU rRNA
**4**[AN]	*Certhiaxis cinnamomeus*					
**12**[BRT]	*Formicivora rufa*					
**81**[AN]	*Cantorchilus leucotis*					
**33**[BRT]	*Tyrannus melancholicus*					
**102**[BH]	*Furnarius rufus*					
**103**[BH]	*Paroaria capitata*	X				
**108**[BRT]	*Chloroceryle americana*	X			X	
**110**[BRT]	*Cantorchilus leucotis*					
**111**[BRT]	*Paroaria capitata*					
**117**[BRT]	*Furnarius leucopus*					
**167**[AN]	*Ramphocelus carbo*					
**244**[AN]	*Cacicus cela*					
**333**[AN]	*Sporophila collaris*	X				
**337**[AN]	*Agelasticus cyanopus*				X	
**341**[AN]	*Furnarius leucopus*	X				
**350**[AN]	*Busarellus nigricollis*			X		
**361**[AN]	*Agelasticus cyanopus*					

**420[BRT]**	*Ramphocelus carbo*		X			X
**441[AN]**	*Pitangus sulphuratus*				X	X
**497[AN/Cal]**	*Saltator coerulescens*		X			X
**507 [AN/Cal]**	*Basileuterus flaveolus*		X			
**516[ANN/EH]**	*Ramphocelus carbo*	X				
**555[HAEM]**	*Leptotila verreauxi*					
**Occurrence for positive controls tested**		5 (21.73%)	3 (13.04%)	1 (4.34%)	3 (13.04%)	3 (13.04%)

[AA]: Stands for primers and probes designed in the present study. The occurrence of each VBA was calculated based on the 40 samples used as positive controls. The following superscripts stand for the next agents *Anaplasma* spp. [AN], *Bartonella* spp. [BRT], *Bartonella henselae* [BH], Haemosporidians [HAEM], '*Candidatus* Allocryptoplasma spp.'[CAI]. Samples indicated with the superscripts were positive in qPCR and PCR obtained from (Alabí Córdova, Fecchio, Calchi, Dias, Machado et al., 2024a; Alabí Córdova, Fecchio, Calchi, Dias, Mongruel et al., 2024a)(Alabí Córdova, Fecchio, Calchi, Dias, Machado et al., 2024a; Alabí Córdova, Fecchio, Calchi, Dias, Mongruel et al., 2024a).. Asterisks indicate the reference from where the primers were obtained. *:[84]

Supplementary Material - Table S3. Results of High-Throughput Microfluidic real-time PCR showing co-positivity for selected vector-borne agents in neotropical birds sampled in Pantanal wetland, Mato Grosso state, central-western Brazil.

**Positive samples on microfluidic PCR**	**Scientific name**	***Anaplasma* spp.**<br>**16S rRNA***	***Bartonella* spp.**<br>***ssrA****	***Plasmodium* spp.[AA]**<br>***cytB***	***Haemoproteus* spp.[AA]**<br>***cytB***	**Onchocercidae filariids. [AA]**<br>**LSU rRNAe**
**Salou4**	*Furnarius rufus*	X				
**Salou9**	*Ramphocelus carbo*					X
**Salou12**	*Synallaxis albilora*					X
**Salou13**	*Crotophaga ani*	X				
**Salou15**	*Pseudoseisura unirufa*	X				
**Salou19**	*Crotophaga ani*	X				
**Salou23**	*Ramphocelus carbo*	X				
**Salou30**	*Ramphocelus carbo*					X
**Salou32**	*Ramphocelus carbo*	X				X
**Salou34**	*Eucometis pennicillata*	X				
**Salou36**	*Ramphocelus carbo*					X
**Salou40**	*Ramphocelus carbo*					X
**Salou41**	*Ramphocelus carbo*	X				X
**Salou42**	*Ramphocelus carbo*					X
**Salou48**	*Myiothlypis flaveola*			X		
**Salou49**	*Ramphocelus carbo*	X				
**Salou51**	*Eucometis pennicillata*	X				

**Salou54**	*Ramphocelus carbo*	X				X
**Salou55**	*Ramphocelus carbo*	X				X
**Salou57**	*Leptotila verreauxi*	X				
**Salou59**	*Synallaxis albilora*	X				
**Salou61**	*Myiothlypis flaveola*	X			X	
**Salou65**	*Ramphocelus carbo*			X	X	
**Salou67**	*Turdus amaurochalinus*	X				X
**Salou70**	*Eucometis pennicillata*	X				
**Salou71**	*Myiothlypis flaveola*	X				
**Salou72**	*Eucometis pennicillata*	X				
**Salou74**	*Hypocnemoides maculicauda*	X				
**Salou77**	*Turdus amaurochalinus*		X			
**Salou78**	*Eucometis pennicillata*	X				
**Salou79**	*Eucometis pennicillata*	X				
**Salou80**	*Synallaxis albescens*	X				
**Salou81**	*Ramphocelus carbo*					X
**Salou82**	*Eucometis pennicillata*	X				
**Salou83**	*Turdus amaurochalinus*	X				
**Salou85**	*Saltator coerulescens*	X				X
**Salou86**	*Cranioleuca vulpine*	X				
**Salou88**	*Dendroplex picus*	X				
**Salou90**	*Icterus pyrrhopterus*	X				
**Salou93**	*Rupornis magnirostris*	X				

**Salou94**	*Ramphocelus carbo*	X				X
**Salou95**	*Fluvicola albiventer*	X		X		
**Salou97**	*Myiothlypis flaveola*	X				
**Salou99**	*Ramphocelus carbo*	X				
**Salou103**	*Ramphocelus carbo*	X				
**Salou105**	*Ramphocelus carbo*	X				
**Salou106**	*Myiothlypis flaveola*	X				
**Salou107**	*Myiothlypis flaveola*	X				
**Salou108**	*Leptotila verreauxi*					X
**Salou114**	*Synallaxis albilora*	X				X
**Salou115**	*Myiophobus fasciatus*	X				
**Salou117**	*Pheugopedius genibarbis*			X		
**Salou121**	*Arremon flavirostris*			X		
**Salou122**	*Taraba major*	X				
**Salou124**	*Synallaxis albilora*					X
**Salou125**	*Ramphocelus carbo*	X		X		
**Salou126**	*Ramphocelus carbo*	X		X		X
**Saolou136**	*Myiophobus fasciatus*	X				
**MIM149**	*Volatinia jacarina*					
**MIM150**	*Myiarchus ferox*	X				
**MIM152**	*Machetornis rixosa*	X				
**MIM153**	*Volatinia jacarina*					
**MIM154**	*Volatinia jacarina*				X	

**MIM166**	*Myiarchus ferox*	X				
**MIM175**	*Ramphocelus carbo*			X		
**MIM176**	*Volatinia jacarina*	X				
**MIM178**	*Columbina talpacoti*				X	
**MIM186**	*Volatinia jacarina*					
**MIM218**	*Columbina talpacoti*				X	
**MIM230**	*Turdus leucomelas*			X		
**MIM234**	*Thraupis palmarum*					
**MIM236**	*Leptotila verreauxi*				X	
**MIM241**	*Volatinia jacarina*			X		
**MIM242**	*Columbina talpacoti*				X	
**MIM243**	*Columbina talpacoti*	X			X	
**MIM250**	*Columbina squammata*				X	
**MIM251**	*Volatinia jacarina*			X	X	
**MIM272**	*Volatinia jacarina*			X		
**MIM278**	*Columbina squammata*				X	
**MIM289**	*Columbina talpacoti*			X	X	
**MIM290**	*Volatinia jacarina*			X		
**MIM292**	*Volatinia jacarina*			X		
**MIM296**	*Volatinia jacarina*			X		
**MIM298**	*Columbina talpacoti*	X			X	
**Total**		50	1	17	13	18

[AA]: Stands for primers and probes designed in the present study. Asterisks indicate the reference from which the primers where obtained *:[84]

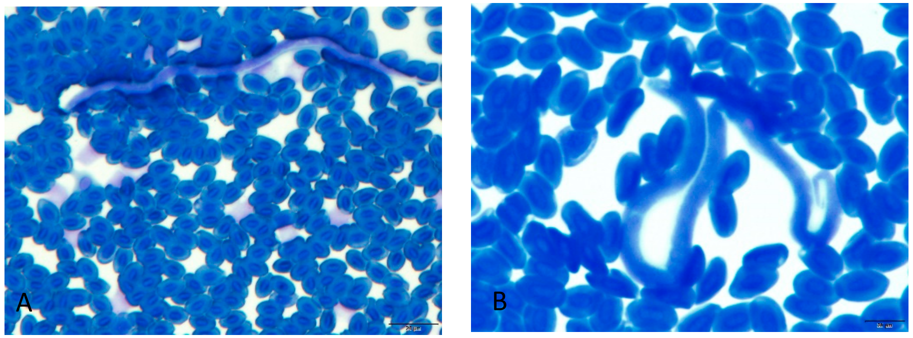

Figure S1. Microfilariae detected in avian sample blood smears. A) SaoLou30 and B) SaoLou41 were identified as *Aproctella* spp. due to sequencing and phylogenetic inference of the *cox-1* gene.
